# Transmission of *Enterobacter aerogenes* septicemia in healthcare workers

**DOI:** 10.1186/s40064-016-3011-x

**Published:** 2016-08-23

**Authors:** Piyush Jha, Choon-Mee Kim, Dong-Min Kim, Jong-Hoon Chung, Na-Ra Yoon, Babita Jha, Seok Won Kim, Sook Jin Jang, Young-Joon Ahn, Jae Keun Chung, Doo Young Jeon

**Affiliations:** 1 Department of Internal Medicine, School of Medicine, Chosun University, 588 Seosuk-dong, Dong-gu, Gwangju, 501-717 Republic of Korea; 2 Department of Neurosurgery, School of Medicine, Chosun University, Gwangju, Republic of Korea; 3 Department of Laboratory Medicine, School of Medicine, Chosun University, Gwangju, Republic of Korea; 4 Premedical Science, College of Medicine, Chosun University, Gwangju, Republic of Korea; 5 Department of Medical Education, School of Medicine, Chosun University, Gwangju, Republic of Korea; 6 Health and Environment Research Institute of Gwangju Metropolitan City, Gwangju, Republic of Korea; 7 Microbiology Division, Jeollanam-do Institute of Health and Environment, Muan, Republic of Korea

**Keywords:** *Enterobacter aerogenes*, Septicemia, Immunocompetent healthcare workers, Gastroenteritis

## Abstract

*Enterobacter aerogenes* is recognized as an important bacterial pathogen in hospital-acquired infections. This report describes two unusual cases of septicemia caused by *E. aerogenes* in immunocompetent healthcare workers. *E. aerogenes* was isolated from blood cultures of the two patients experiencing septicemia. The clinical isolates were initially identified as *E. aerogenes* using a VITEK II automated system and 16S rRNA sequence analysis, and; both isolates involved in the outbreak shared a common pulse-field gel electrophoresis pattern. The similarities between the two cases included the simultaneous development of gastroenteritis symptoms, severe sepsis and thrombocytopenia after taking intravenous injections of ketorolac tromethamine. A common source of normal saline, a 100 mL bottle, was used for diluting the analgesic in both cases. In addition to the general population, healthcare workers, especially those who are also intravenous drug abusers, should be considered subjects that could cause a transmission of *Enterobacter* infection.

## Background

*Enterobacter* is a genus of the family Enterobacteriaceae, consisting of common Gram-negative, facultative anaerobic, rod-shaped, non-spore-forming bacteria. *E. aerogenes* is recognized as an important bacterial pathogen in hospital acquired infections (Jarvis and Martone 1992). In this study, we examined the transmission of *E. aerogenes* infections in two healthcare workers; by isolating the pathogen from the blood cultures of both the patients and complete genomic sequence analysis and pulsed-field gel electrophoresis (PFGE) revealing that both the isolates were identical.

*Enterobacter* is the eighth most common pathogen in healthcare-associated infections in the United States (Hidron et al. 2008) and constitutes 2.9 % of healthcare-associated bloodstream infections in Korea (Son et al. 2010). We encountered two patients who were otherwise healthy nurses with no underlying conditions and had nearly identical clinical signs and symptoms 2 h apart, transferred to our Emergency department from the same hospital. The transmission of *E. aerogenes* in health care workers has not been reported anywhere until now; thus this report describes the first transmission of *E. aerogenes* in healthcare workers.

## Methods

### Case description

We encountered two patients in our Emergency department on 17th October, 2013 with nearly identical clinical signs and symptoms who were otherwise healthy nurses with no underlying conditions. Both the patients were admitted 2 h apart with complaints of multiple episodes of vomiting and loose stool. They also complained of pain in the epigastric and right upper quadrant (RUQ) regions. On examination, the abdomen was soft but tender in the RUQ region, blood pressure (BP) was below the normal range, and heart rate and respiratory rate were elevated. The findings of the physical examinations of both patients were unremarkable. The laboratory findings of both patients are presented in Table 1. Blood culture was performed; meanwhile, initial treatment was started with intravenous fluids, metronidazole and ceftriaxone. Arterial blood gas (ABG) analysis revealed metabolic acidosis in one patient (Case 1). The blood culture turned positive on the 2nd hospital day.

**Table 1:** Laboratory findings of the two cases

Case	Age/sex	Laboratory investigation findings								Co-infection
		WBC (counts/mm^3^)	Hemoglobin (g/dL)	Platelet counts (mm^3^)	Liver function test				Serum creatinine (mg/dL)	
					T. Bil (mg/dL)	AST (U/L)	ALT (U/L)	S. Alb (g/dL)		
Case 1	41/F	3960	11.6	60,000	2.55	122	278	3.10	2.61	Hepatitis C virus *Burkholderia cepacia*
Case 2	37/F	3480	12.9	53,000	3.97	256	210	2.88	1.52	–

*WBC* white blood cell, *T. Bil* total bilirubin, *AST* aspartate aminotransferase, *ALT* alanine aminotransferase, *S. Alb* serum albumin

After receiving the blood culture reports, which were positive for *E. aerogenes* in both patients, we investigated the two cases in detail, as we suspected a common source of *E. aerogenes* infection. The drug resistance patterns of the two isolated *E. aerogenes* strains were identical; resistant to ampicillin/sulbactam, intermediately resistant to imipenem.

The two patients were not aware that they had experienced similar symptoms until after they were admitted to our hospital. During the investigation, we found that the two patients had used the same saline bottle to prepare ketorolac injections that they then self-administered, and that neither patient was aware of the other.

Metronidazole was stopped after completion of a 5-day course, and ceftriaxone was replaced with cefepime on the 8th hospital day after we got culture positive result. The general condition of both patients improved gradually, and all biochemical parameters returned to within their normal ranges before the patients were discharged, which was after 2 weeks of hospitalization.

Informed written consent was obtained from both the patients for participation in this study.

### Antimicrobial susceptibility testing

The clinical isolates were identified with a VITEK II automated system (bioMe´rieux, Marcy l’Etoile, France). Antimicrobial susceptibility determinations including the MICs were performed automatically with the VITEK II system.

The MIC was interpreted as susceptible or resistant according to the guidelines of the Clinical and Laboratory Standards Institute (CLSI) MIC interpretive standards for *E. aerogenes* where applicable (Gouby et al. 1994; National Committee for Clinical Laboratory Standards 2003a, b).

### Bacterial isolates and media

*Enterobacter aerogenes* was isolated from the blood cultures of both patients with sepsis. The organisms were cultivated in LB broth (Difco Laboratories, Detroit, MI, USA) at 37 °C for 12-18 h with agitation. Nutrient agar (Eiken Chemical, Tokyo) and 5 % sheep blood agar (Becton–Dickinson, Tokyo) were the solid media used for *E. aerogenes* culture. Strains of *E. aerogenes* were stored at −80 °C in 3 % skim milk (Difco) supplemented with 5 % glucose (Difco).

### Polymerase chain reaction (PCR) and sequencing

Molecular identification of two *E. aerogenes* isolates was performed using conventional PCR (C-PCR) targeting the 16S rRNA gene. Genomic DNA was extracted from bacterial cultures using the QIAamp DNA mini kit (Qiagen, Westburg, Netherlands) according to the manufacturer’s instructions. C-PCR was performed in a 20 µL reaction volume using the primers 27F (5′-AGAGTTTGATCCTGGCTCAG-3′; *Escherichia coli* 16S ribosomal DNA base pair positions 8–27) and 1492R (5′-TACGGHTACCTTGTTACGACTT-3′, positions 1492–1507). Each reaction mixture contained AmpliTaq Gold^®^ 360 Master Mix (Applied Biosystems, Waltham, MA, USA), 1 µL each of the 5 µM forward and reverse primers, and 2 µL of genomic DNA. The cycling conditions consisted of the following steps: 2 min at 95 °C; 30 cycles of 1 min at 95 °C, 30 s at 55 °C, and 45 s at 72 °C; and a 10 min extension at 72 °C. The PCR products were visualized by electrophoresis on an ethidium bromide-stained 1.5 % agarose gel. A Biosystems Veriti™ 96-Well Thermal Cycler (Applied Biosystems, Foster City, CA) was used for this experiment. Amplified and purified DNA was prepared for direct sequencing using a QIAquick PCR Purification Kit (Qiagen, Westburg, Netherlands) and was sequenced by dideoxy termination with an automatic sequencer (ABI Prism 3730XL DNA analyzer). Sequence homology analysis was performed by the National Center for Biotechnology Information (National Institutes of Health) BLAST network service, and both of our sample species had 99 % pairwise similarity with the complete genome of *E. aerogenes* KCTC 2190 (accession no. CP002824).

### Pulsed-field gel electrophoresis of isolates

**Fig. 1:**
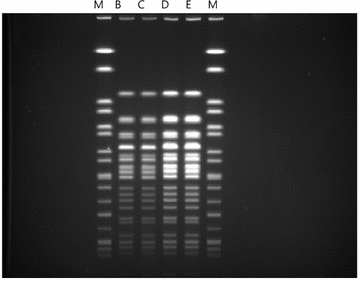
Pulsed-field gel electrophoresis (PFGE) profiles of *E. aerogenes* from humans after Xba-I digestion. *Lane M*: markers (*Salmonella braenderup* ATCC BAA-664), *Lanes B, C*: case 1 and *Lanes D, E*: case 2

We also performed pulsed-field gel electrophoresis (PFGE) to confirm that the two *Enterobacter* species isolated from the patients were the same strain of the bacterium. The two *E. aerogenes* isolates were subjected to DNA restriction analysis with 10 U/µl of the SmaI enzyme in appropriate buffer. The DNA fragments were separated by pulsed-field gel electrophoresis through a 1.2 % agarose gel as described previously (Murchan et al. 2003). We could document the identical DNA banding patterns based on typing results (Fig. 1).

## Results

*Enterobacter aerogenes* was isolated from the blood cultures of both patients. Complete genomic sequencing analysis revealed that both the isolates were identical. PFGE also showed that both the strains were indistinguishable (Fig. 1).

The drug resistance patterns of the two isolated *E. aerogenes* strains were identical. They were resistant to ampicillin/sulbactam (MIC ≥ 2 µg/mL) and were intermediately resistant to imipenem (MIC ≥ 2 µg/mL). They were susceptible to amikacin (MIC < 2 µg/mL), ceftriaxone (MIC < 1 µg/mL), tigecycline (MIC < 0.5 µg/mL), gentamicin (MIC < 1 µg/mL), piperacillin (MIC < 4 µg/mL) colistin (MIC < 2 µg/mL), cefepime (MIC < 1 µg/mL), ceftazidime (MIC < 2 µg/mL), ciprofloxacin (MIC 0.25 µg/mL), meropenem (MIC < 0.25 µg/mL), minocycline (MIC < 1 µg/mL) and piperacillin/tazobactam (MIC < 4 µg/mL).

Both patients were managed successfully, and all biochemical parameters returned to within their normal ranges within 2 weeks of hospitalization, at which time the patients were discharged.

## Discussion

*Enterobacter aerogenes*, a component of the normal flora of the human gastrointestinal tract, is a significant nosocomial pathogen and a common cause of iatrogenic bacteremia (Hidron et al. 2008). The incidence of bacteremia due to *E. aerogenes* has increased gradually, accounting for nearly 11 % of nosocomial infections in some series (Acolet et al. 1994; Lin et al. 2006). Although community-acquired infections are occasionally observed, nosocomial infections are, the most frequent by far. Patients most susceptible to acquire *Enterobacter* infections are those who stay in the hospital, especially in the intensive care unit (ICU) for prolonged periods. Other major risk factors for *Enterobacter* infections include the prior use of antimicrobial agents; concomitant malignancy (especially hemopoietic and solid organ malignancies); hepatobiliary disease; ulcers of the upper gastrointestinal tract; diabetes mellitus; chronic renal failure; and immunosuppression (Lin et al. 2006).

The gastrointestinal tract is a common endogenous reservoir for *E. aerogenes,* and spread of infection from the gastrointestinal tract is difficult to ascertain, which may explain why the portal of entry of *E. aerogenes* often cannot be identified (Kanemitsu et al. 2007). Outbreaks have been traced to various common sources, including total parenteral nutrition solutions, isotonic saline solutions, albumin, digital thermometers, intravenous catheters, mechanical ventilator and dialysis equipment.

In both of our patients, sepsis was severe and had initial symptoms of gastro-enteritis, and the same *E. aerogenes* strain was isolated in the blood of both patients. Our first patient also had a co-infection with *Burkholderia cepacia*, a member of a bacterial group known as the *B. cepacia* complex. This infection mainly occurs in patients with underlying lung disease, such as cystic fibrosis and chronic granulomatous disease, and in immunocompromised individuals, hospitalized patients and drug addicts (Govan et al. 1996). A limitation of this study is that we could not find the residual saline solution, used for diluting ketorolac tromethamine prior to intravenous injection, as it had already been discarded before we began investigating the source of *E. aerogenes* in these two cases.

A peculiarity of our cases is that both patients developed severe sepsis preceded by gastroenteritis symptoms after self-administration of intravenous ketorolac mixed with normal saline taken from a common source at the hospital where they worked. We do not know exactly how the normal saline solution was contaminated with *Enterobacter.* Although it is evident that *Enterobacter* strains commonly arise from endogenous intestinal flora of hospitalized patients and that a nurse’s hand can be contaminated with *Enterobacter* while taking care of patients, this incident highlights the possibility of hospital-acquired sepsis due to traditional nosocomial microorganisms in drug abusers who are also healthcare workers and have access to hospital supplies. Neither of these patients had any history of any severe underlying diseases, prior antimicrobial use or any previous hospital admission.

## Conclusion

Here, we conclude that in addition to the general population, healthcare workers, especially those who are also intravenous drug abusers, should be considered as subjects who could be a source of transmission of pathogens like *E. aerogenes*.

## References

[CR1] Acolet D, Ahmet Z, Houang E, Hurley R, Kaufmann ME (1994). Enterobacter cloacae in a neonatal intensive care unit: account of an outbreak and its relationship to use of third generation cephalosporins. J Hosp Infect.

[CR3] Gouby A, Neuwirth C, Bourg G, Bouziges N, Carles-Nurit MJ, Despaux E, Ramuz M (1994). Epidemiological study by pulsed-field gel electrophoresis of an outbreak of extended-spectrum β-lactamase producing *Klebsiella pneumoniae* in a geriatric hospital. J Clin Microbiol.

[CR4] Govan JR, Hughes JE, Vandamme P (1996). *Burkholderia cepacia*: medical, taxonomic and ecological issues. J Med Microbiol.

[CR5] Hidron AI, Edwards JR, Patel J, Horan TC, Sievert DM, Pollock DA, Fridkin SK (2008). NHSN annual update: antimicrobial-resistant pathogens associated with healthcare-associated infections: annual summary of data reported to the National Healthcare Safety Network at the Centers for Disease Control and Prevention, 2006–2007. Infect Control Hosp Epidemiol.

[CR6] Jarvis WR, Martone WJ (1992). Predominant pathogens in hospital infections. J Antimicrob Chemother.

[CR7] Kanemitsu K, Endo S, Oda K, Saito K, Kunishima H, Hatta M, Inden K, Kaku M (2007). An increased incidence of *Enterobacter cloacae* in a cardiovascular ward. J Hosp Infect.

[CR8] Lin YC, Chen TL, Ju HL, Chen HS, Wang FD, Yu KW, Liu CY (2006). Clinical characteristics and risk factors for attributable mortality in *Enterobacter cloacae* bacteremia. J Microbiol Immunol Infect.

[CR9] Murchan S, Kaufmann ME, de Ryck ADR, Struelens M, Zinn CE, Fussing V, Salmenlinna S, Vuopio-Varkila J, Solh NE, Cuny C, Witte W, Tassios PT, Legakis N, van Leeuwen W, van Belkum A, Vindel A, Laconcha I, Garaizar J, Haeggman S, Olsson-Liljequist B, Ransjo U, Coombes G, Cookson B (2003). Harmonization of pulsed-field gel electrophoresis protocols for epidemiological typing of strains of methicillin-resistant *Staphylococcus aureus*: a single approach developed by consensus in 10 European laboratories and its application for tracing the spread of related strains. J Clin Microbiol.

[CR10] National Committee for Clinical Laboratory Standards (2003a) Performance standards for antimicrobial disk susceptibility tests, 8th edn. M2-A8. National Committee for Clinical Laboratory Standards, Wayne, PA

[CR11] National Committee for Clinical Laboratory Standards (2003b) Methods for dilution antimicrobial susceptibility tests for bacteria that grow aerobically, 6th edn. Approved Standard M7-A6. National Committee for Clinical Laboratory Standards, Wayne, PA

[CR12] Son JS, Song JH, Ko KS, Yeom JS, Ki HK, Kim SW, Chang HH, Ryu SY, Kim YS, Jung SI, Shin SY, Oh HB, Lee YS, Chung DR, Lee NY, Peck KR (2010). Bloodstream infections and clinical significance of healthcare-associated bacteremia: a multicenter surveillance study in Korean hospitals. J Korean Med Sci.

